# Assessing the Association of Sodium, Potassium Intake and Sodium/Potassium Ratio on Blood Pressure and Central Adiposity Measurements amongst Ellisras Undernourished, Rural Children Aged 5–13 Years: South Africa

**DOI:** 10.3390/children9030422

**Published:** 2022-03-16

**Authors:** Thato Tshepo Raphadu, Moloko Matshipi, Peter Modupi Mphekgwana, Kotsedi Daniel Monyeki

**Affiliations:** 1 Department of Physiology and Environmental Health, University of Limpopo, Sovenga 0727, South Africa; thatoraphadu@gmail.com (T.T.R.); moloko.matshipi@ul.ac.za (M.M.); 2 Department of Research Administration and Development, University of Limpopo, Sovenga 0727, South Africa; peter.mphekgwana@ul.ac.za

**Keywords:** sodium, potassium, sodium/potassium ratio

## Abstract

Background: Numerous studies have shown how diet, such as sodium (Na) and potassium (K) intake, is an important risk factor for non-communicable diseases (NCDs). This study aimed to assess the relationship between sodium intake, potassium intake; and sodium/potassium ratio with blood pressure (BP) and abdominal obesity amongst Ellisras rural children. Method: In this cross-sectional study, data on dietary intake of sodium and potassium were collected using a 24-h recall questionnaire from a total of 765 participants, aged 5–13 years. Blood pressure and anthropometric measurements were also collected. Generalised linear models and Pearson correlation were conducted to assess the association of sodium intake, potassium intake; and their ratio with BP, waist circumference (WC), and waist-to-height ratio (WHtR). Results: In both age groups, less than 14.9% of males and 19.8% of females consumed above the recommended adequate intake (AI) of sodium. In addition, both age groups had more than 90% of males and females who consumed below the recommended AI of potassium. Moreover, the sodium/potassium ratio was above the WHO recommended level in more than 30% of males and females. The study found a significant, weak positive correlation of sodium intake with systolic BP (SBP), diastolic BP (DBP), and with WHtR. A significant, weak positive correlation was also found between sodium/potassium ratio and WHtR. In addition, a significant association was found between potassium intake and systolic BP. Conclusion: Although our study found a notable low average intake of sodium and potassium as compared to the recommended values. There was positive correlation found between sodium intake and BP. Furthermore, a positive correlation of sodium intake and sodium/potassium ratio with WHtR was also found.

## 1. Introduction

World Health Organisation (WHO) has reported that non-communicable diseases (NCDs) are the main contributors to mortality and morbidity in the world [1], killing 41 million each year, which is equivalent to 71% of deaths [2]. Evidence reported by various researchers has shown diet as an important risk factor for NCDs. Furthermore, the prevalence of NCDs has greatly and continuously increased over the years [3,4]. Non-communicable diseases that can be predicted by high levels of sodium and low level of potassium are hypertension, obesity, cardiovascular diseases (CDVs), cancer, and osteoporosis [4,5,6]. Although these diseases are more prevalent in adults, there has been a growing concern as these diseases continue to be detected in children and adolescents [7,8].

A progressive increase in BP in childhood or young adulthood through middle age, may predict the level of BP and the presence of hypertension later in life [5]. Elevated levels of sodium intake and inadequate levels of potassium intake may affect the development of hypertension. High consumption of potassium, commonly from fruits and vegetables, such as bananas, potatoes, and spinach, can counteract the negative effects of high sodium intake on BP [1]. Dietary habits developed from childhood into adulthood have indicated that children with an extreme level of sodium and lower potassium intake tend to maintain those levels over time [8]. Although the association of sodium and potassium intake has only been shown concerning BP, recently there have been few studies suggesting a link regarding sodium intake in relation to body weight in children and adolescents independent of total energy intake [9,10,11,12]. However, the collective effect of dietary sodium and potassium intake in the pathogenesis of other conditions, including overweight and obesity, especially in children; is less known [13]. Moreover, there is a crucial need to educate the public to keep a low dietary sodium intake and adequate level of potassium in children as diet in childhood can play a significant role in determining adult dietary habits [14].

Institutions worldwide have designed dietary guidelines in their national food and nutrition policies [15,16]. These guidelines are designed to curb the increase in NCDs by reducing sodium intake and increasing potassium intake in the population, as it has been identified as one of the most cost-effective strategies to reduce NCDs [4]. A study by He et al. (2008) showed that minimising the source of sodium and increasing the source of potassium may lead to reducing the health complications associated with BP [14]. As a means to maintain a healthy level of BP over the progression of life, there are strong initiatives that tried to find a way to reduce sodium intake among children, mainly in Europe and North America [17,18,19]. Despite the initiative to reduce sodium and emphasise the importance of potassium, few studies in Africa and South Africa have explored the trends of sodium and potassium intake and their health effects in cross-sectional or longitudinal studies. Studies conducted in South Africa and Africa on sodium and potassium intake have consistently focused on adults, and not on children [15]. These studies have shown high content of sodium and low content of potassium. In addition, data from studies conducted in children are limited, especially those assessing both sodium and potassium intake; and their ratio in health outcomes [1]. Currently there are no studies in South Africa that have looked at the effect of sodium intake, potassium intake and sodium/potassium ratio on hypertension and obesity in children.

Undernutrition is one of the most important public health issue, which has a prevalence of more 900 million people around the world. It is responsible for the highest death rate in children and has long-lasting physiological effects, which includes increased susceptibility to fat accumulation mostly in central region of the body and hypertension [20]. A previous study by Van Den Ende et al. (2014) focused on the relationship between dietary intake and body mass index (BMI) among the same sample (children), whilst Mashiane et al. (2018) focused on the same sample when they were young adults in Ellisras [21,22]. Van Den Ende et al. (2014) reported a high prevalence of underweight (undernourished) amongst the same sample [21]. However, Mashiane et al. (2018) revealed a high prevalence of obesity and overweight as the Ellisras sample grow older [22]. The increase in overweight and obesity in populations where historical undernutrition prevailed is often characterised by a pattern of nutrition transition [23]. However, this study is a cross-sectional study which focused on investigating the relationship of daily intake of sodium, potassium intake, and the daily dietary sodium/potassium ratio with blood pressure and central adiposity measurements amongst Ellisras rural children.

## 2. Method and Materials

### 2.1. Sampling Procedure

This study is part of the ongoing Ellisras longitudinal study (ELS), of which the details of the geographical area were reported elsewhere [24]. The ELS initially followed a cluster sampling method. Briefly, the study was undertaken at 22 schools (10 pre-school and 12 primary schools) randomly selected from 68 schools within the Ellisras area [24]. A baseline data that were collected in 1996, with follow-up exams of dietary intake, BP and anthropometric measurements from 1999 [24]. However, this study deployed a cross-sectional study design in which information on dietary intake was collected from Ellisras in 1999. A total of 765 participants (394 males and 371 females), aged 5–13 years participated in this study.

### 2.2. Exclusion Criteria

Any participant with the following was excluded from this study:

Participants who failed to provide a signed consent form before measurements.

### 2.3. Dietary Intake

Data on diet were collected using a 24-h recall questionnaire [25]. Trained ELS field workers interviewed participants regarding their dietary intake over the past 24 h. Parents/caregivers of all the participants were interviewed regarding the dietary intake over the previous 24 h. The estimated portion size of foods consumed were recorded in as much detail as possible, using a pre-tested questionnaire and food models simulating average portions of local foods. An average of two days of dietary intake was taken for each participant. One dietary intake was collected during the weekday and another dietary intake for the weekend. This is because food consumption during the weekdays differs from the food consumed during the weekend. People tend to eat a lot more during the weekend than during the week, especially on a Saturday [26]. The average of sodium intake and potassium intake were analysed using local food tables and the South African Food Composition Database System (SAFOODS) [27] and were compared with the recommended intakes for sodium and potassium as described by Consensus Study Report (2019) (Table 1) [28]. The WHO recommends a sodium/potassium ratio of 1:1 (or ≤1) to assess the average molar sodium/potassium ratio [1]. Since the recommended sodium/potassium ratio is in moles, the sodium and potassium intake in this study were converted from milligrams to milli-moles. The following conversion was used to calculate the average molar sodium/potassium ratio [29]:

23 mg sodium = 1 mmol sodium;

39 mg potassium = 1 mmol potassium.

### 2.4. Anthropometric and Blood Pressure Measurements

Blood pressure was measured using an electronic Micronta monitoring kit, at least three blood pressure readings were taken after the participant had been seated for 5 min, as described by the National High Blood Pressure Education Program Working Group on Hypertension Control in Children and Adolescents [30]. The cut–off points for high blood pressure or hypertension as described by National Heart, Lung, and Blood Institution (NHLBI) [31] was utilised for age, sex, and height. Blood pressure was calculated using the Merck manual-medical calculator for both boys and girls separately (Merck manual, 2018) (Table 1). Weight, height, and waist circumference were measured according to standard procedures of the International Society for the Advancement of Kinanthropometry (Norton and Olds, 1996) [32]. The waist-to-height ratio was calculated as WC divided by height in centimetres. Abdominal obesity was defined as waist circumference and waist-to-height ratio (Table 1).

### 2.5. Statistical Analysis

IBM SPSS Statistics software package (version 26.0) was used to conduct statistical analysis. To describe and characterise the samples, descriptive statistics were calculated for all the variables to indicate frequencies (expressed as percentages) and median. All the variables were stratified by age groups and sex. Shapiro–Wilk test was used to assess variables normality. Parametric (one-way ANOVA), Chi-square, and Fisher’s exact tests were conducted to determine the difference between males and females, stratified by age groups (4–8 years and 9–13 years) in all variables. Generalised linear models and Pearson correlation were conducted to assess the association of sodium intake, potassium intake; and their ratio with BP, WC and WHtR. The probability value for statistical significance for all tests was set at a *p*-value ≤ 0.05.

## 3. Ethical Clearance

Ethical clearance was granted by the Turfloop Research Ethics Committee (TREC) of the University of Limpopo before the study was conducted. This study is part of the ongoing ELS that started in 1996 with ethical clearance number MREC/P/204/2013:IR.

## 4. Results

### 4.1. Characteristics of the Population

This study constitutes 765 participants (394 males and 371 females). In the age group 4–8 years, the mean values of sodium intake were 507.34 mg/d for males and 553.70 mg/d for females. The mean values of potassium intake were 1041.03 mg/d for males and 1020.53 mg/d for females. Furthermore, the mean values of the sodium/potassium ratio for males and females were 0.89 and 1.00, respectively. The mean values of WC for males and females were 52.19 cm and 51.70 cm; and 0.43 and 0.42 for WHtR, respectively. The mean values of SBP and DBP were 97.10 mmHg and 60.91 mmHg for males and 97.00 mmHg and 60.63 mmHg for females, respectively. Among the age group 4–8 years, there were no significant mean differences between males and females.

In children aged 9–13 years, the mean values of sodium intake for males and females were 550.03 mg/d and 598.20 mg/d, whilst the mean values of potassium intake for males and females were 1027.77 mg/d and 1190.11 mg/d, respectively. The mean values of the sodium/potassium ratio were 1.00 and 0.99 for males and females, respectively. In addition, the mean values of WC for males and females were 55.94 cm and 56.25 cm. However, the mean value of WHtR was the same for both males and females, which was 0.40. The mean values of SBP and DBP were 100.77 mmHg and 62.12 mmHg for males and 102.93 mmHg and 62.91 mmHg for females, respectively. There were no statistical mean differences between males and females in children aged 9–13 years except for potassium intake, height and SBP shown in Table 2.

### 4.2. The Prevalence of Sodium, Potassium Intake, Sodium/Potassium Ratio, Hypertension and Abdominal Obesity according to WC and WHtR

In children aged 4–8 years, 14.9% of males and 19.8% of females consumed above the recommended adequate intake (AI) of sodium, whilst 98.5% of males and 99.0% of females consumed below the recommended AI of potassium. The sodium/potassium ratio was above the WHO recommended ratio in 38.1% of males and 45.5% of females. In addition, the prevalence of hypertension according to high systolic and diastolic blood pressure was 13.4% for males and 6.9% for females. The prevalence of abdominal obesity according to WHtR for males was 0.7% and 2.0% for females. The Chi-square and Fisher’s exact test p-values for all the variables were more than 0.05, suggesting that there was no significant difference in the sample proportions between males and females in Table 3.

In children aged 9–13 years, 10.0% of males and 13.0% of females consumed above the recommended AI of sodium, whilst 99.2% of males and 93.2% of females consumed below the recommended AI for potassium, respectively. Moreover, the sodium/potassium ratio was above the WHO recommended ratio in 34.6% of males and 39.6% of females. The prevalence of hypertension according to high systolic and diastolic blood pressure was 3.0% for males and 7.7% for females. The abdominal obesity according to WC prevalence in males was 60.0% and 56.3% in females. The prevalence of abdominal obesity according to WHtR for males was 0% and 0.4% for females. Since a vast majority were underweight (undernutrition) in a study conducted by Van Den Ende et al. (2014) on the same participants [21], the high prevalence of WC as compared to WHtR might due to bloating of malnourishment, rather than visceral fat. The Chi-square or Fisher’s exact test *p*-values for potassium intake and hypertension according to high diastolic and systolic blood pressure were less than 0.05, suggesting that there is a significant difference in the sample proportions between males and females in the 9–13 years age group in Table 3.

### 4.3. The Correlation of Sodium, Potassium Intake and with Systolic Blood Pressure, Diastolic Blood Pressure, Waist Circumference and Waist-to-Height Circumference

Results in Table 4 indicates a significant, weak positive correlation of sodium intake with SBP (*r* = 0.192, *p*-value = 0.026), DBP (*r* = 0.185, *p*-value = 0.031), and with WHtR (*r* = 0.176, *p*-value = 0.041) in males aged 4–8 years. In addition, a significant, weak positive correlation was found between sodium/potassium ratio and WHtR (*r* = 0.184, *p*-value = 0.034) in the same age group of males.

### 4.4. The Effect of Sodium, Potassium Intake and Sodium/Potassium Ratio on Systolic and Diastolic Blood Pressure, Waist Circumference and Waist-to-Height Circumference

Results in Table 5 indicate a decrease in potassium intake [***β*** = 0.102, (95% CL: 0.004, 0.200), *p*-value = 0.028] was associated with an increase in SBP. Even when the data were adjusted for age and sex, potassium intake [***β*** = 0.090, (95% CL: −0.007, 0.187), *p*-value = 0.038] was still associated with SBP.

Table 6 shows the most frequent food items used in Ellisras rural children aged 5–13 years. This food list is placed in an order from most used to least used food item.

## 5. Discussion

The purpose of this study was to assess the association between sodium intake and potassium intake; and their ratio with BP, WC, and WHtR. Across two days of dietary intake, in children aged 4–8 years the mean (average) of sodium intake for males were 507.34 mg/d and 553.70 mg/d for females, whilst in children aged 9–13 years the median values of sodium intake for males and females were 550.03 mg/d and 598.20 mg/d. The mean values of sodium intake in both age groups (4–8 and 5–13 years) were notably lower than the recommended AI. A study conducted by Van Den Ende et al. (2014) on the same sample reported a significant large percentage of this population were underweight, while the prevalence of overweight and obesity was very low [21]. This might explain why the levels of sodium intake were notably lower than the recommended. Although that might be the case, our findings were inconsistent compared to similar studies conducted in Morocco, Europe, America, and China, whereby their average intakes were notably higher than the recommended intake. The average of sodium in the Moroccan study was 2235.3 mg/d [4], whilst the average of sodium intake in European countries among children was between 2400 mg/d and 3000 mg/d [33,34,35,36]. In addition, America and China reported the highest average of sodium intake at 3100 mg/d and 3400 mg/d [37,38]. This difference might be due to the differences in a geographical area, demographics, and socioeconomic status. Since this study was conducted in a rural area and previous research has shown that the traditional eating habits of most South Africans residing in rural areas consist mostly of a prudent diet which consists of adequate content of sodium and potassium [22,39,40,41,42]. This might account for the reason of having a low average of sodium in this population compared with other countries, such as Europe, America, China, and Morocco.

On the other hand, the mean of potassium intake was 1041.03 mg/d for males and 1020.53 mg/d for females in children aged 4–8 years, whilst in children aged 9–13 years the mean of potassium intake for males and females was 1027.77 mg/d and 1190.11 mg/d. This might be due to different food intake. The mean of potassium intake in both age groups were notably lower than the recommended AI. However, it is often challenging to compare values from studies on children especially between boys and girls, mainly due to the different nutritional requirements, particularly when considering potassium recommendations depending on energy needs [43]. Our consumed average intake of potassium in this study, amongst males and females in both age groups were, respectively, lower compared with studies conducted by Campanozzi et al. (2015) and Oliveria et al. (2015) [33,44]. The estimated average of potassium intake was 1530 mg/d in boys and 1400 mg/d in girls in a study conducted by Campanozzi et al. (2015) [33]. In addition, Oliveria et al. (2015) reported that the average of boys and girls were, respectively, 1701.0 mg/d and 1682.0 mg/d [44].

Furthermore, the mean of the sodium/potassium ratio in children aged 4–8 years was 0.89 for males and 1.00 for females. The mean of the sodium/potassium ratio was 1.00 and 0.99 for males and females. The mean of sodium/potassium ratio in both age groups were below or equal to the WHO recommended ratio of ≤1. The low average intake of sodium and potassium might have contributed to sodium/potassium ratio being below or equal to the WHO recommended ratio. In comparison with similar studies, the average of sodium/potassium ratio in our study was similar with the finding of a study conducted in North America, but was notably lower as compared to studies conducted in Europe. For instance, the average of sodium/potassium ratio reported in American children is 1.03 [45]. In addition, the average of sodium/potassium ratio in Spanish children (aged 6–14 years) and French children (aged 2–14) were, respectively, 3.6 and 1.64 [46,47]. Countries in Europe did not comply with the recommended sodium/potassium ratio.

Moreover, in our study 14.9% of males and 19.8% of females in children aged 4–8 years; and 10.0% of males and 13.0% of females in children aged 9–13 years consumed above the recommended AI of sodium. A study conducted in Morocco evaluated sodium intake in a population aged 6–18 years, and have shown that sodium intakes were too high and found 41.2% of boys and 58.8% of females consumed above the recommended AI of sodium [4]. In our study, a total of 98.5% in males and 99.0% in females consumed below the recommended AI of potassium in children aged 4–8 years. At the same time, in children aged 9–13 years, 99.2% of males and 93.7% of females consumed below recommended AI of potassium. These findings are similar to those reported in Italy, where over 96% of boys and 98% of girls consumed lower than the recommended adequate intake of potassium [33]. In our study a total of 35.1% of males and 45.5% of females consumed above WHO recommended sodium/potassium ratio in children aged 4–8 years. At the same time, in children aged 9–13 years, 34.6% of males and 39.6% of females consumed above WHO recommended sodium/potassium ratio.

A significant, weak positive correlation of sodium intake with SBP, DBP, and with WHtR in males aged 4–8 years. In addition, a significant, weak positive correlation was found between sodium/potassium ratio and WHtR in the same age group of males. A decrease in potassium intake was associated with an increase in BP (SBP). Even when the data were adjusted for age and sex, potassium intake was still associated with SBP. Over the years, studies have shown that in children, an increase in BP is associated with high dietary sodium intake [48,49] and reduced BP is associated with lower dietary sodium intake [50]. Evidence on the effect of potassium and sodium intake on BP (SBP or DBP) in children is mixed [19]. Some studies have found a positive correlation between sodium, potassium intake and sodium/potassium ratio with BP [51,52], whilst others have not [53,54,55,56,57]. In our study, there were no significant correlation found among sodium, potassium intake, and sodium/potassium ratio with blood pressure (SBP or DBP) and abdominal obesity (WC and WHtR) in children aged 4–9 years, in females only and in children aged 9–13 years (both males and females). In addition, an increase in sodium intake and a decrease in potassium did not affect the development of abdominal obesity (WC and WHtR) in this population. Few epidemiological studies have indicated a positive correlation of high sodium and low potassium with adiposity measures [18,19]. Diets high in sodium and low in potassium are often high in energy and, therefore, may promote weight gain which may also lead to overweight or obesity [1,13].

As a result of a global situation with excessive levels of sodium intake and deficient levels of potassium intake [4], WHO has developed and implemented global strategies and effective policies for salt consumption, targeting the main sources of dietary sodium intake for all age groups [2]. Due to this situation, South Africa has taken the stand to become the first country in the world to regulate sodium consumption at the manufacturing level for several industries. These new regulations are designed to reduce the level of sodium in certain food products [58].

The main strength of this study could be that we used interviewer-administered questionnaires, which are more effective than self-administered questionnaires [39]. However, a 24-h recall could be considered as a limitation. Because 24-h urinary excretion of sodium and potassium has been considered to be the “golden standard’’ method of obtaining data on sodium and potassium intake in population surveys and more accurate than the 24-h recall questionnaire [4,59]. Although this research is part of an ongoing research, the fact that the data for research was collected in 1999 may serve as a methodological limitation of this present study.

## 6. Conclusions

In our study, we found that this population has a low average intake of sodium and potassium. However, a significant, weak positive correlation of sodium intake with SBP, DBP and with WHtR, and also a significant, weak positive correlation was found between sodium/potassium ratio and WHtR. In addition, a decrease in potassium intake had an effect on the increase in SBP. However, more research is needed to further examine how dietary patterns of sodium and potassium can serve as predictors of hypertension and abdominal obesity in South African and African children especially over time; so that those consistent conclusions can be drawn regarding the status of sodium and potassium in children.

## Figures and Tables

**Table 1:** Classification of variables according to sex and age.

Variables	Life Stage Group (Years)			
	Male 4–8 Years	Female 4–8 Years	Male 9–13 Years	Female 9–13 Years
Na adequate intake (mg/d)	≤1000	≤1000	≤1200	≤1200
K adequate intake (mg/d)	≥2300	≥2300	≥2500	≥2300
Na/K ratio	≤1	≤1	≤1	≤1
Hypertension (systolic and/or diastolic)	>95th percentile	>95th percentile	>95th percentile	>95th percentile
Abdominal obesity according to WC	≥90th percentile	≥90th percentile	≥90th percentile	≥90th percentile
Abdominal obesity according to WHtR	≥0.5	≥0.5	≥0.5	≥0.5

**Table 2:** Descriptive characteristics of the population according to sex and age.

Life Stage Group (Years)	Male 4–8 Years (*n* = 134)	Female 4–8 Years (*n* = 101)	One-Way Anova *p*-Value	Male 9–13 Years (*n* = 260)	Female 9–13 Years (*n* = 270)	One-Way Anova *p*-Value
Variables	Mean ± SD	Mean ± SD		Mean ± SD	Mean ± SD	
Na (mg/d)	507.34 ± 578.60	553.70 ± 530.312	0.459	550.03 ± 988.10	598.20 ± 654.51	0.556
K (mg/d)	1041.03 ± 584.66	1020.53 ± 540.19	0.844	1027.77 ± 721.58	1190.11 ± 1020.53	0.032 *
Na/K ratio (mmol/mmol)	0.89 ± 0.95	1.00 ± 0.96	0.282	1.00 ± 1.20	0.99 ± 0.91	0.996
Weight (Kg)	20.69 ± 2.97	20.80 ± 2.97	0.743	28.12 ± 4.36	29.06 ± 5.33	0.024 *
Height (cm)	122.31 ± 7.10	122.99 ± 6.43	0.428	138.79 ± 7.57	139.77 ± 7.66	0.167
WC (cm)	52.20 ± 2.94	51.71 ± 3.24	0.225	55.94 ± 3.47	56.25 ± 4.35	0.370
WHtR	0.43 ± 0.02	0.42 ± 0.26	0.052	0.40 ± 0.02	0.40 ± 0.02	0.661
SBP (mmHg)	97.10 ± 11.20	97.00 ± 11.60	0.542	100.77 ± 9.90	102.93 ± 10.92	0.017 *
DBP (mmHg)	60.91 ± 9.98	60.63 ± 9.33	0.832	62.12 ± 8.52	62.91 ± 9.59	0.318

* *p*-value < 0.05; n—number of individuals. Na—sodium, K—potassium, Na/K ratio—sodium/potassium ratio, SBP—systolic blood pressure, DBP—diastolic blood pressure, mg/d—milligram per day, mmol/d—millimole per day.

**Table 3:** The prevalence of sodium, potassium intake, sodium/potassium ratio, hypertension and abdominal obesity according to WC and WHtR.

Life Stage Group (Years)	Male 4–8 Years (*n* = 134)	Female 4–8 Years (*n* = 101)	Chi-Square/Fisher’s Exact Test *p*-Value	Male 9–13 Years (*n* = 260)	Female 9–13 Years (*n* = 270)	Chi-Square/Fisher’s Exact Test *p*-Value
Variables	*n* (%)	*n* (%)		*n* (%)	*n* (%)	
Na-Proportion > 1000 (4–8 years) mg/d or >1200 mg/d (9–13 years)	20 (14.9)	20 (19.8)	0.305	26 (10.0)	35 (13.0)	0.305
K-Proportion < 2300 mg/d or <2500 mg/d	132 (98.5)	100 (99.0)	0.444	258 (99.2)	253 (93.7)	0.017 *
Na/K- proportion > 1	51 (38.1)	46 (45.5)	0.238	90 (34.6)	107 (39.6)	0.208
Hypertension according to high SBP and DBP	18 (13.4)	7 (6.9)	0.639	8 (3.0)	21 (7.8)	0.025 *
Abdominal obesity according to WC	19 (14.2)	12 (11.9)	0.622	156 (60.0)	152 (56.3)	0.388
Abdominal obesity according to WHtR	1 (0.7)	2 (2.0)	0.400	0 (0)	1 (0.4)	0.326

* *p*-value < 0.05; n—number of individuals. Na—sodium, K—potassium, Na/K ratio—sodium/potassium ratio, WC—waist circumference, WHtR—waist-to-height ratio, SBP—systolic blood pressure, DBP—diastolic blood pressure, mg/d—milligram per day. AI—adequate intake.

**Table 4:** The correlation of sodium, potassium intake and with SBP, DBP, WC, and WHtR in Pearson correlation.

Variables	Na		K		Na/K	
Male 4–8 Years	*r*	*p*-Value	*r*	*p*-Value	*r*	*p*-Value
SBP (mmHg)	0.192	0.026 *	0.152	0.079	0.073	0.400
DBP (mmHg)	0.185	0.031 *	0.160	0.063	0.006	0.948
WC (cm)	−0.042	0.632	−0.069	0.426	0.004	0.960
WHtR	0.176	0.041 *	−0.013	0.877	0.184	0.034 *
Female 4–8 years						
SBP (mmHg)	0.095	0.342	−0.034	0.733	0.100	0.317
DBP (mmHg)	0.169	0.092	−0.079	0.433	0.166	0.097
WC (cm)	−0.052	0.607	−0.016	0.874	−0.050	0.620
WHtR	−0.065	0.521	−0.059	0.555	−0.048	0.634
Male 9–13 years						
SBP (mmHg)	0.046	0.461	0.039	0.531	0.075	0.227
DBP (mmHg)	0.089	0.155	0.043	0.494	0.098	0.113
WC (cm)	0.063	0.309	0.014	0.820	0.030	0.629
WHtR	−0.016	0.803	0.051	0.414	−0.052	0.408
Female 9–13 years						
SBP (mmHg)	0.011	0.859	0.012	0.0838	0.049	0.426
DBP (mmHg)	0.022	0.715	0.057	0.350	0.043	0.480
WC (cm)	0.075	0.221	0.059	0.338	0.042	0.496
WHtR	0.095	0.121	−0.015	0.807	0.100	0.100

* *p*-value < 0.05; Na-sodium, K-potassium, Na/K ratio- sodium/potassium ratio, SBP-systolic blood pressure, DBP-diastolic blood pressure, WC-waist circumference, WHtR- waist-to-height ratio, mg/d- milligram per day, mmol/d- millimole per day, cm-centimeter, *r*-correlation coefficient.

**Table 5:** Regression coefficients (*β)* and 95 % confidence intervals (CI) of the generalised linear model.

Variables	Na			K			Na/K			Na*K		
Unadjusted	*β*	95% CL	*p*-Value	*β*	95% CL	*p*-Value	*β*	95% CL	*p*-Value	*β*	95% CL	*p*-Value
SBP (mmHg)	0.025	−0.126, 0.175	0.746	0.102	0.004, 0.200	0.028 *	0.074	−0.053, 0.200	0.254	−0.031	−0.072, 0.009	0.126
DBP (mmHg)	0.058	−0.093, 0.208	0.453	0.079	−0.019, 0.177	0.115	0.047	−0.080, 0.174	0.466	−0.020	−0.060, 0.020	0.330
WC	0.015	−0.136, 0.166	0.844	0.028	−0.071, 0.126	0.580	0.018	−0.109, 0.145	0.789	0.010	−0.031, 0.226	0.634
WHtR	0.117	−0.034, 0.267	0.130	−0.008	−0.106, 0.091	0.877	−0.049	−0.176, 0.178	0.451	−0.036	−0.077, 0.004	0.077
Adjusted for age and sex												
SBP (mmHg)	0.040	−0.108, 0.187	0.599	0.090	−0.007, 0.187	0.038 *	0.058	−0.066, 0.183	0.358	−0.036	−0.075, 0.004	0.077
DBP (mmHg)	0.064	−0.086, 0.214	0.401	0.074	−0.024, 0.172	0.140	0.040	−0.086, 0.167	0.531	−0.022	−0.062, 0.018	0.282
WC	0.049	−0.086, 0.183	0.478	0.011	−0.077, 0.099	0.812	−0.013	−0.127, 0.100	0.817	−0.002	−0.038, 0.034	0.903
WHtR	0.087	−0.052, 0.226	0.221	0.011	−0.080, 0.103	0.805	−0.020	−0.137, 0.097	0.740	−0.027	−0.064, 0.011	0.160

* *p*-value < 0.05; Na—sodium, K—potassium, Na/K ratio—sodium/potassium ratio, SBP—systolic blood pressure, DBP—diastolic blood pressure, WC—waist circumference, WHtR—waist-to-height ratio, mg/d—milligram per day, mmol/d—millimole per day, ***β***—beta coefficient and confidence intervals (CI).

**Table 6:** The most frequent used food items in children aged 5–13 years in Ellisras.

Maize Porridge or Sorghum Porridge
Tea
Sugar (white)
Brown
Homemade bread
Chicken
Spinach
Non dietary creamer
Beef
Red meat (from goat and wild animals)
Tomato and onion
Cooked dry beans
White bread
Margarine
Fried egg
Fish (canned pilchard or fresh from Lephalale)
Sorghum beer (homemade)
Cooked cabbage
Cold drink (mostly Coke)
Peanut butter
White rice
Sweets
Mashontja (Mopani worms)
Bananas and oranges
Cow milk
Jam

Source adapted [21].

## Data Availability

Data will be made available upon request.
